# The Impact of Escaped Farmed Atlantic Salmon (*Salmo salar* L.) on Catch Statistics in Scotland

**DOI:** 10.1371/journal.pone.0043560

**Published:** 2012-09-06

**Authors:** Darren M. Green, David J. Penman, Herve Migaud, James E. Bron, John B. Taggart, Brendan J. McAndrew

**Affiliations:** Institute of Aquaculture, University of Stirling, Stirling, Stirlingshire, United Kingdom; Institute of Marine Research, Norway

## Abstract

In Scotland and elsewhere, there are concerns that escaped farmed Atlantic salmon (*Salmo salar* L.) may impact on wild salmon stocks. Potential detrimental effects could arise through disease spread, competition, or inter-breeding. We investigated whether there is evidence of a direct effect of recorded salmon escape events on wild stocks in Scotland using anglers' counts of caught salmon (classified as wild or farmed) and sea trout (*Salmo trutta* L.). This tests specifically whether documented escape events can be associated with reduced or elevated escapes detected in the catch over a five-year time window, after accounting for overall variation between areas and years. Alternate model frameworks were somewhat inconsistent, however no robust association was found between documented escape events and higher proportion of farm-origin salmon in anglers' catch, nor with overall catch size. A weak positive correlation was found between local escapes and subsequent sea trout catch. This is in the opposite direction to what would be expected if salmon escapes negatively affected wild fish numbers. Our approach specifically investigated documented escape events, contrasting with earlier studies examining potentially wider effects of salmon farming on wild catch size. This approach is more conservative, but alleviates some potential sources of confounding, which are always of concern in observational studies. Successful analysis of anglers' reports of escaped farmed salmon requires high data quality, particularly since reports of farmed salmon are a relatively rare event in the Scottish data. Therefore, as part of our analysis, we reviewed studies of potential sensitivity and specificity of determination of farmed origin. Specificity estimates are generally high in the literature, making an analysis of the form we have performed feasible.

## Introduction

Since the industry began in the 1960s, production of farmed Atlantic salmon (*Salmo salar* L.) in the North Atlantic gradually increased to reach 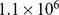 tonnes in 2009, while annual catch of Atlantic wild salmon has decreased from *c.* 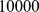 to 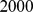 tonnes over the same period [1]. There is concern regarding the large size of the farmed stocks relative to wild fish, particularly over potential adverse impacts of escaped farmed salmon through potential interbreeding with wild fish. In Scotland alone, 1.9 million farmed salmon escaped into the natural environment between 2002–9 [2]. Potential detrimental effects could include increased infestation by sea lice [3], competition for food or other resources, and inter-breeding enabling the spread of farmed genes into the wild population [4], thereby potentially lowering fitness [5], [6]. Counteracting these processes, the breeding success of escaped farmed salmon appears low [5]. Escapes can occur at any point in the production cycle from the rearing of juveniles to the smolt stage in fresh water, through ongrowing to marketable size in the sea. Conceivably, with niche overlap between brown trout (*Salmo trutta* L.) and Atlantic salmon, especially in juvenile stages, these competitive effects could extend inter-species. However, there is evidence that brown trout are the more dominant fish [7], potentially reducing this impact. Potential escape routes include storm damage, or holes in nets and cages both in freshwater and seawater. Routes for escapes and the resulting consequences have been recently reviewed by [8] and [9].

Scottish wild salmon catch has dropped in recent years in farmed areas, coinciding with the rise in salmon farming, located primarily on the west coast, however this is mirrored by a parallel decline in eastern regions without salmon farming, and the rod count alone has remained similar on both coasts (see results section below for examination of the publically available recent data). Rod count for sea trout (the anadromous form of brown trout, *Salmo trutta* morpha *trutta*) has suffered greater decline on the west coast, but this decline predates the establishment of salmon farming there. There may well be confounding factors not taken into account when comparing the East with the West of Scotland through such summary statistics; however, suspicion remains that salmon farming may be a partial cause of the decline. Though our study concerns escapes of farmed salmon, there are several potential mechanisms by which salmon farming could impact wild salmon without this being mediated by escapes, for example, by a rise in the density of sea lice in sea lochs [10]. A significantly higher percent of rod catch reported as farmed salmon in rivers with salmon farms in their sea lochs has been noted [11], alongside reduced freshwater salmon populations in rivers with salmon farms in their mouths [11]. However, such correlation data are insufficient to demonstrate cause and effect.

The River Ewe (Scotland) has been the focus of detailed study, with both salmon farming and a high level of reported escapes in the catch statistics [11], [12]. Reported local escapes occurred in 1989 (marine growers), 1990 (smolts), 1992 (a large number of parr and smolts), 1993 (growers) and not again until 1999 (growers, parr, smolts). This matches poorly with the reported rod catch of farmed salmon, which peaked in 1995 and 1997, with lower counts in 1993, 1994 and 1999 [12]. Total rod catch in the Ewe catchment in recent years (including reported farmed salmon) is also within the range experienced prior to the establishment of salmon farming in that catchment. A wider study of monitoring and reporting of escaped farmed salmon in the British Isles found no association between reported escapes and the prevalence of escapes in coastal and freshwater fisheries, and also a weak association between farm production and the prevalence of escapes [13]. Nevertheless, these authors aggregated their data at a regional level, and suggested that a finer geographical scale of study is warranted, as we respond to in the current study.

Some of these previous studies suggested but did not prove links between catch statistics and salmon escapes. Analysis of these data sources is complicated by the potential for confounding factors, and most would not allow effects of salmon escapes *per se* to be distinguished from general effects of salmon farming. Therefore, in this paper, we address a very specific question: whether or not documented escape events can be linked statistically to later changes in catch statistics, either in terms of overall catch, or in terms of the proportion of the catch that are reported as being escaped farmed salmon. Sea trout remain in coastal waters, more directly exposed to potential environmental effects of marine aquaculture, therefore we also analysed the sea trout catch statistics. As a counterpart to this analysis, we considered the likely data quality of catch statistics in terms of accuracy of reporting of the farmed *versus* wild origin of salmon. Our analysis is up to date, using the recently available data on catch and escapee numbers. As a result, our perception of the current trends in salmon and sea trout catch differs somewhat from what would have been concluded even a few years ago.

## Materials and Methods

### Data sources

**Figure 1:**
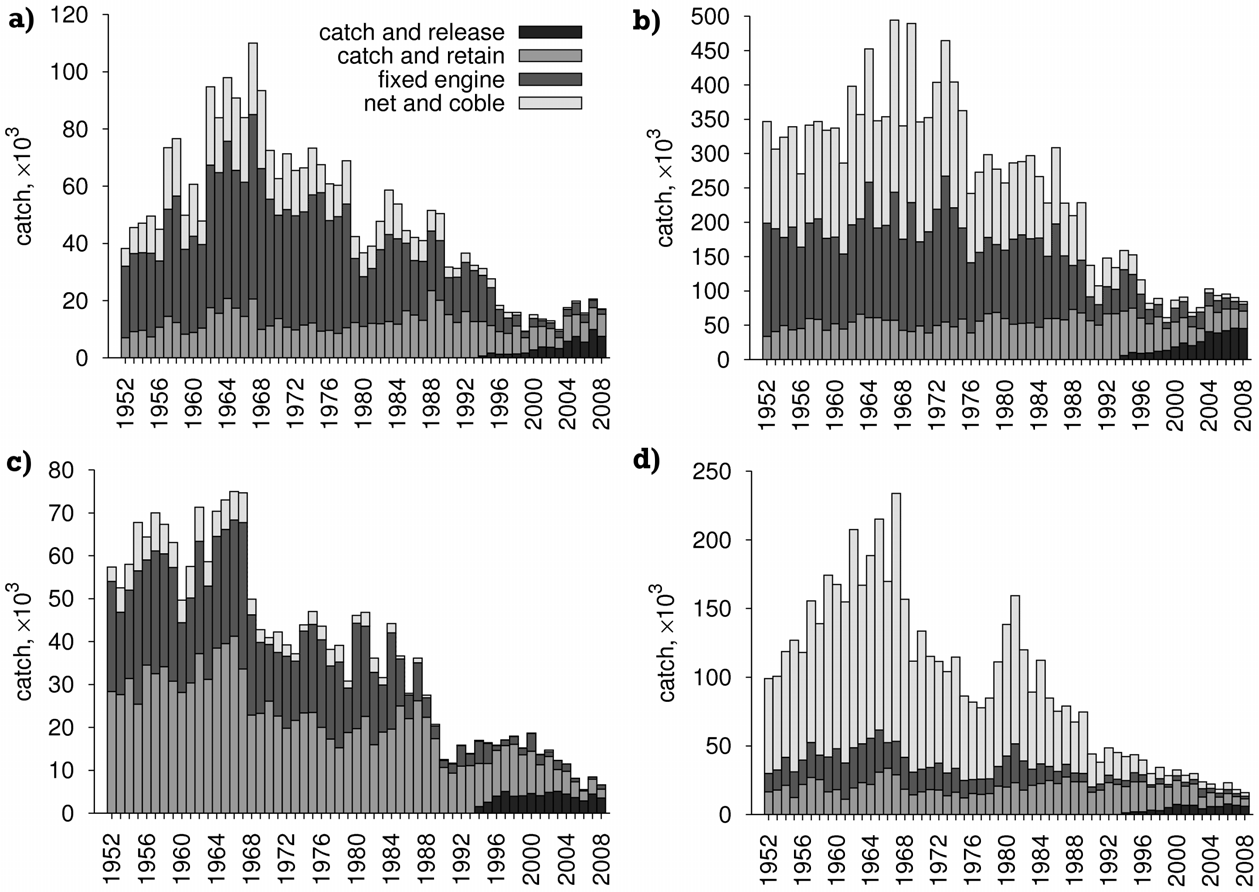
Historical catch data for salmon and sea trout in Scotland. a) west coast salmon; b) east coast salmon; c) west coast sea trout; d) east coast sea trout. East coast: Cape Wrath to Berwick (not including the Northern Isles); west coast: Solway Firth to Cape Wrath plus the Northern Isles. Data with permission from Marine Scotland Science (see Acknowledgements).

**Figure 2:**
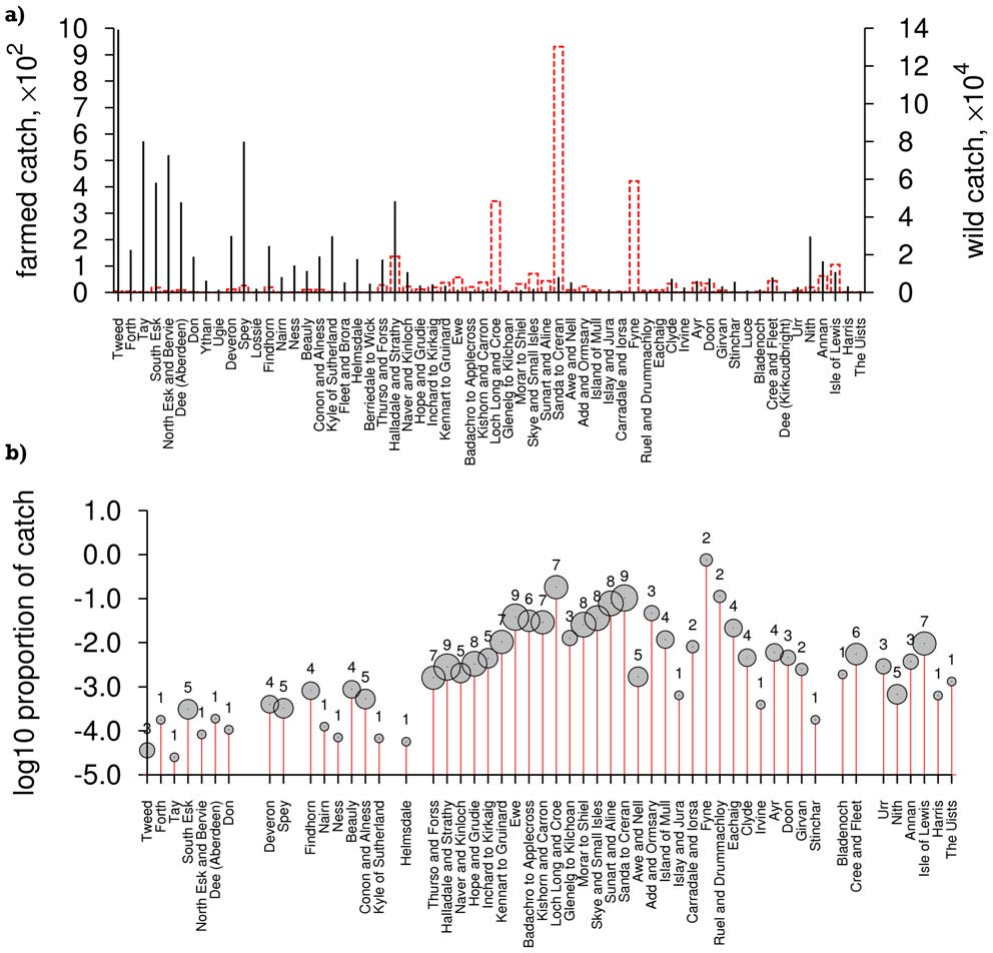
Catch statistics by district (2001 to 2009 data, excluding the Northern Isles). a) Dashed boxes, left axis: farmed catch; lines, right axis: wild catch. b) Proportion of catch of farmed origin, with symbol size indicating number of years (out of 9) excluding districts without catch of salmon of farmed origin. Data with permission from Marine Scotland Science (see Acknowledgements).

**Figure 3:**
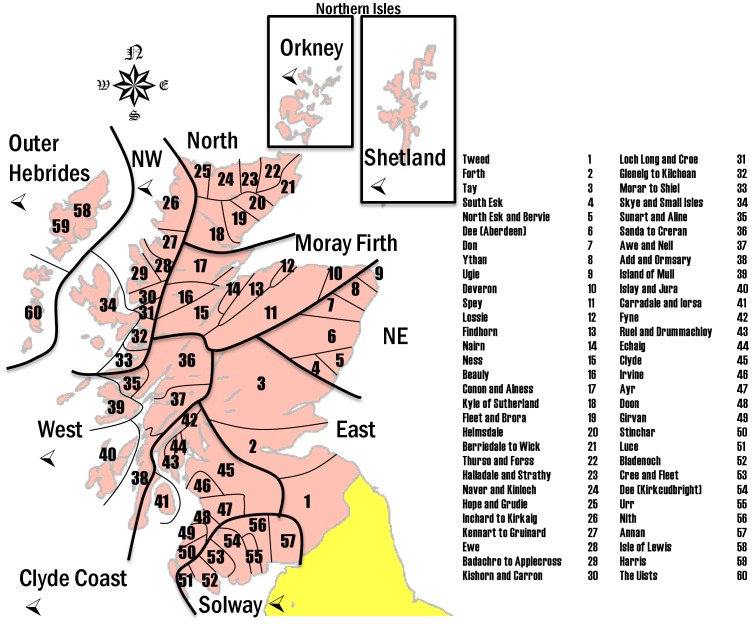
Schematic map of fisheries statistics districts and regions in Scotland. Regions regarded as ‘west coast’ in the results section are indicated by a left-pointing arrowhead. ‘Districts’ and ‘regions’ are not coterminous with other political units of similar name in UK geography.

Historic (Fig. 1) and recent (2001 to 2009 [14]; Fig. 2) catch data were tabulated against 62 salmon fishery statistical districts in 11 salmon fishery statistical regions (pooled as east and west coasts, Fig. 3). This includes all salmon (including grilse, i.e. salmon returning to freshwater after one winter) caught by rod and line (both retained and released), net and coble (sweep netting using small boats), or fixed engine (e.g. various types of nets, often specific to a local area); and for both wild and farmed caught salmon. The definition of these four catch methods are documented by the Scottish Government [15] and focus on different parts of the water course: fixed-engine fisheries are coastal, outside estuary limits, whereas net-and-coble fisheries may operate in estuaries and lower river reaches. The largest fraction of catch is accounted for by rod-and-line angling, predominantly above tidal limits. Rod-and-line angling is divided into ‘catch and retain’ and ‘catch and release’, with the latter becoming an increasingly large proportion of the take for both trout and salmon, as catch size has reduced.

With catch and release (widely implemented in Scotland for salmon since the 1990s, as a conservation tool), there is potential for double counting, but with time-trends in the balance between caught and retained and caught and released, it was assumed pooling the counts was more robust. Catch data for Orkney and Shetland were sparse and these regions were excluded from further analysis. Catch data for sea trout were treated similarly, with sufficient data for Shetland also included. With trout, effects of identifiable farmed salmon escape events can be studied without potential misidentification of wild (trout) with farmed (salmon).

Reporting of escapes for farmed salmon is mandatory (since 2001), and the available data consisted of count, date, size, and location of escapes by farm name. Escape counts were summed across each statistical district over each calendar year from 2002 to 2009. Additional variables consist of the escapes data lagged by between one to five years, to test for a delayed effect of salmon escapes. Data for both (lagged) escapes *and* catch were available for 2007 to 2009.

### Analysis

Two types of models were constructed. In the *proportion escapes* models (1), the proportion of catch 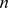 for each district–year consisting of farmed salmon 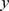 was regressed against year 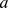, region 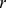, and district 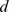, plus the the incidence of recent escaped farmed salmon in the same district 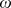, including lag terms. In the *catch statistics* models (2), the total catch per district–year (for both salmon and trout) was related to the same factors and covariates. Models were built using the R software environment, using binomial errors for the proportion escapes model, and Poisson errors for the catch statistics model. Likelihood ratio tests were used to compare nested models; each model was ordered with escape terms later in the list of terms, so as to specifically test for a significant effect of escapee salmon over and above any other local effects. For ease of interpretation, McFadden's pseudo r-square statistics are presented below.
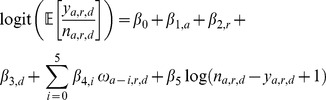
(1)

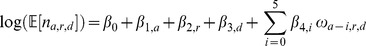
(2)For the *proportion escapes* model alone (1), 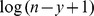 was fitted as a covariate as a proxy for (otherwise unknown) fishing effort.

As a test of model robustness, a related ANOVA model was fitted in both cases, with appropriate transformation of data. For the proportion escapes model, a weighted least-squares fit was performed on the empirical logit, with response variable
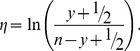
(3)The weighting variable used was the reciprocal of the variance [16], estimated as
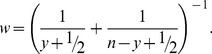
(4)Explanatory variables were the same as in the generalised linear models.

Each model was fitted to data at the level of the statistical district. Nevertheless, with little evidence of how far escaped salmon disperse, models were also fitted at the statistical region level. As it is unclear which life stages are most likely to impact on wild salmon, models were fitted using either all escapes, or only large marine salmon (over 500 g). Models were also built excluding eastern Scotland, without an active salmon farming industry in the marine stage.

## Results

### Escape and catch statistics: historic and recent data

Examining the historical catch statistics for the east and west sides of Scotland, where the east side has minimal marine salmon aquaculture, a similar overall long-term downwards trend can be seen in catch statistics (Fig. 1). Some recovery of catch size for salmon can be seen in the last five years on both coasts, though the decrease in sea trout catch on both coasts shows no such halt. An important caveat with these data is the lack of any measure of fishing effort. Nevertheless, there is little sign of an increase in rod catch consequent to declining commercial catch effort, which is a major contributor to the overall decline.

For recent catch data (2001–9), 0.30% of overall catch was identified as of farmed origin, with a higher proportion (2.8%) within the intensely farmed regions (West, North West, Clyde Coast, Outer Hebrides; Fig. 3). The highest catch of farmed-origin salmon was in the West region (5.8%), and the lowest in the East region (0.0045%), where there is no farming activity at all.

From 2002–9, 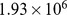 escaped salmon were reported across Scotland in 100 escape events, with considerable geographical (Fig. 2) and annual variation in numbers, from 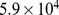 in 2008 to 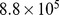 in 2005. Of these, 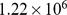 were large salmon (>500 g) at sea, in 77 escape events. Overall, there was no significant correlation between the nationwide proportion of salmon catch reported as farmed, and the numbers of escaped salmon in that or the two preceding years (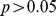). For older salmon (as opposed to grilse), catch of farmed-origin fish was stratified into two periods: January to April, and May to December. 95% of farmed-origin salmon were reported in the latter period.

### Anglers' ability to distinguish farmed-origin salmon

The catch data used in this study are of unknown accuracy, specifically with regards to the specificity and sensitivity of the anglers' ability to identify farmed salmon. To clarify this, reports from the literature [4], [12], [17]–[22] were examined to attempt to estimate these parameters. One study [4] sampled salmon from the River Polla (Scotland), known to contain farmed and wild salmon. These were categorised as putative wild or escaped on the basis of morphology. Carotenoid pigment analysis agreed with this categorisation, with 65 of 65 fish with fin deformities containing canthaxanthin, and 14 of 14 fish without such deformities only containing astaxanthin. Bankside assessment of wild/farmed state was of similar success rate, with 18 of 18 wild fish, and 26 of 26 farmed fish correctly categorised as farmed or wild. In another study in the River Ewe (Scotland) [12], 95 of 95 wild salmon, and 7 of 10 farmed salmon were correctly identified. And using scale characteristics, a further study [18] confirmed 100 of 101 fish initially classed as of reared origin to have been correctly classified. Several other papers have commented on the difficulty of categorising salmon origin. A study in Greenland produced two datasets [19]: in one, 3 of 272 fish were identified as farmed, but 7 were uncategorisable on the basis of scales; in the second, 6 of 423 fish were identified as farmed, with 6 difficult to categorise. A similar problem was reported in Faroese data [20], [21] where 6% of fish were found to be uncategorisable. A Norwegian study [22] compared scale readings with fishers' initial assessment of farmed/wild origin of salmon. They found that 4 of 7 fish initially assessed as wild were correctly reported, as were 373 of 378 fish initially reported as of farmed origin. For a review of papers exploring various morphological and biochemical methods to detect salmon of farmed origin, see [17].

Sensitivity and specificity estimates are presented in Table 1 with binomial confidence intervals obtained from the *binom.profile* function in R. These studies indicate high specificity for detecting farmed-origin fish, though with wide confidence intervals where sample size is restricted. Sensitivity estimates are also high. High specificity supports the 2001–8 Scottish catch statistics, where some regions report vanishingly low proportions of farmed salmon, however it is unsafe to assume that attribution of fish origin is consistent across districts.

**Table 1:** Sensitivity and specificity estimates for anglers' ability to discern origin of caught salmon.

Reference	true 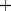	false 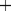	false 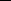	true 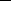	sensitivity	specificity
[4]	65	0	0	14	1 (0.95–1)	1 (0.80–1)
…	26	0	0	18	1 (0.88–1)	1 (0.84–1)
[12]	7	0	3	95	0.70 (0.39–0.91)	1 (0.97–1)
[22]	373	5	3	4	0.99 (0.97–0.99)	0.44 (0.17–0.75)
[18]	100	1	n/a	n/a	n/a	n/a
[19]	3	<7		262	>0.7	>0.96
…	6	<6		411	>0.5	>0.99
[21]	n/a	<6%		n/a	>0.94	

Positive = farmed fish. 95% confidence intervals are provided.

### Models for proportion of escapes

In all models of the proportion of escapes, district and region were highly significant. For the district level model, all regions, 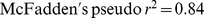. When included in this model, year of study was significant in a likelihood ratio test (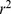 = 0.87). Inclusion of the term for log catch size did not cause a significant reduction in deviance. Including counts of large escapes (0–5-year lags) caused a significant reduction in deviance (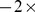 log-likelihood:(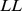)) from 459 to 324 (6 d.f.). This was a better fit than including all escaped salmon (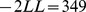) though with a small difference in deviance.

All these effects are relatively small compared with the null-model deviance of 3626, but significant given the large size of the dataset. In this model, of the individual lag terms, only two were significant in a Wald test (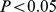), and with contrasting signs. The zero-year term had a coefficient of 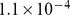, suggesting a relative odds of a caught salmon being identified as of farmed origin of 3.0 for each 10,000 escaped salmon; The four-year lag term had a coefficient of 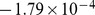, suggesting a relative odds of 0.16 for each 10,000 escaped salmon. The other lag terms were both insignificant and of inconsistent signs. The equivalent ANOVA model indicated the lag terms to be significant overall (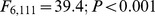) but a small contributor to overall variance and without any individual lag terms significantly different from zero.

In the regional-level models, region and year remained highly significant factors (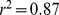), as was the covariate term for catch size, if included (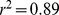). The best fit model included the counts of all escaped salmon and their lags (as opposed to large salmon alone), causing a significant reduction in deviance from 271 to 34. All lag terms except the 5-year lag were significant, and all were positive. That with the largest coefficient was for a one-year lag (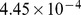). Only the zero-year lag term was significant in the equivalent ANOVA, though all lag terms were of similar magnitude and the same sign as in the logistic regression.

For large escapes, district-level models were repeated for active farming areas only (Outer Hebrides, West, North West, Clyde Coast). Model results were similar to the all-Scotland model in that the zero-year lag term was significantly positive (coef. 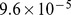), and the four-year lag term significantly negative (coef. 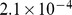). In addition, the one-year lag term was also significantly negative (coef. 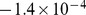). As with the all-Scotland model, the equivalent ANOVA model did not identify any lag terms as significantly different from zero.

The prevalence of farmed-origin salmon across the four different catching methods varied, with higher prevalence in fixed engine and net and coble take (24.8% in west-coast salmon farmed regions) compared with rod and line (1.1%), potentially confounding the analyses given geographical and temporal variation in catch methods. Therefore, we fitted models separately to rod-and-line and ‘other’ fishing methods (which had a 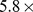 higher prevalence of farmed-origin salmon overall) at the regional level for actively farmed regions and large escapes. Catch-method data at the district level were not available. Repeating the earlier analysis for regional-level data and large escaped fish for rod-and-line catch only, escapes remained significant in a likelihood ratio test (deviance reduction from 112 to 29, compared with a null deviance of 1560). All coefficients for escape terms were significantly negative (largest coefficient, four-year lag, 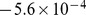), except that for the zero-year lag which was significantly positive (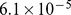). For catch other than rod-and-line, escapes were significant under a likelihood ratio test but no coefficients were significant according to the Wald test.

Deviance residuals were examined to investigate goodness of fit. For the district-level model (all districts, large escaped salmon only), the deviance residuals showed a peaked distribution that deviated from normality (A–D test, 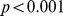). This distribution resulted from districts in the dataset having no reports of caught farmed fish over the whole period, resulting in districts with zero residual. Removing these districts led to a complicated result, though residuals showed a more normal distribution. The model using escapes of all salmon was of similar likelihood to that of large escapes only (deviance of 127.0 versus 129.9). The deviance attributed to escapes in both models was significant and similar (103 versus 100 compared with a null deviance of 2167). However, only in the all-escapes model were escape terms significant in a Wald test (all except one-year-lagged escapes), with all coefficients positive, the largest of which was associated with a four-year lag (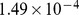).

### Models for catch size

In the Poisson regression of salmon catch size at the district level, region and district were highly significant (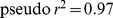), explaining as might be expected the majority of the model deviance, because catches differ greatly between districts; including year caused a significant improvement in model fit (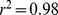). Including numbers of large escapes (plus lag terms) caused a significant reduction in deviance from 7926 to 7618 (6 d.f.), a larger reduction compared to including escapes of all sizes (residual deviance 7759). Only the one-year lag term was significant, with a coefficient of (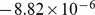) corresponding to a decrease in catch in a district–year of 8.4% per 10,000 escaped salmon. The lag terms were not significant in the equivalent ANOVA model.

As with the proportion of escapes models, the regional-level catch size model gave conflicting results. Region was highly significant (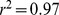), and year significantly improved the model fit on inclusion (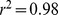). All escape terms were negative and significant, with the highest coefficient for the two-year lag term (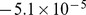). As with the district-level model, these terms were insignificant in the related ANOVA model.

The same models were fitted for trout catch data, and in both region-level and district-level models, terms accounting for escapes of farmed salmon of all sizes produced a model with a higher likelihood than large escapes alone. Again, region, district, and year were highly significant, reflecting variability in trout catch (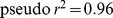). All terms for escaped salmon were significant when included, reducing model deviance from 6782 to 6081; however, they were not all of like sign: All except the zero-year-lag term were positive, the largest being that for the four-year lag (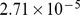), and that for the zero-year lag being 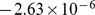. The regional-level model gave similar results with coefficients of like sign. As with the other model types, the equivalent ANOVA model indicated fewer significant lag-escape terms, but where significant these were of like sign and similar magnitude to the Poisson regression model.

A significant number of deviance residuals from the Poisson regression in excess of two were found. As a result, an alternate model was fitted with negative binomial errors. For all districts and large escaped salmon, this model proved a better fit (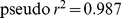) with the majority of residuals in the range 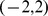. Inclusion of terms for escapes were not significant in a likelihood ratio test (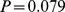).

## Discussion

Recaptures reported above account for less than two per thousand of reported escapes, with the fate of the vast majority of escapes unknown. This suggests that escaped salmon either have very low survival in the wild, disperse without returning, or are less readily caught by anglers. Few studies have examined this in Scotland. However, after a simulated escape by the release in 2006 of 678 tagged adult salmon near Ullapool, only five tags were retrieved: two detached, on beaches in Scotland north of the release site, and three on live fish in Scandinavia [23]. It has been hypothesised that escaped salmon in Scotland move east in this way as a combination of instinctive homing behaviour and prevailing current direction [23].

This contrasts with the situation in Norway, where recapture rate of released cultured salmon has been shown to reach as high as 67% [24]. The difference may be in part due to topographical differences between Scotland and Norway, where enclosed fjords exist at much larger sizes than the west coast of Scotland. However, much of this recapture of escaped farmed salmon occurred in Norway in coastal waters, not rivers [22], and recent data show these fish to perform relatively poorly with low survival to maturity due to impaired feeding [25], and loss of migratory performance [26]. Nevertheless after simulated escape of farmed smolts and post-smolts in Norway, tagged fish were recovered after up to three winters at sea [27], though these were small in number compared with those recaptured more quickly, and across wide area of both river (26%) and sea, albeit with the majority close to the site of release.

Our data source does not indicate the distance from river mouth where farmed- and wild-origin fish are caught, that is whether farmed-origin fish are more or less likely to penetrate to the upper reaches. However, there is a strong trend towards a higher prevalence of farmed-origin salmon in fixed-engine and net-and-coble catch (lower down the water course) than in rod-and-line (further up the water course). As a proportion of overall total catch, salmon of farmed origin are comparatively uncommon compared with similar studies in both Norway [28] and eastern North America (Canada and USA) [29], although more comparable if only the non rod-and-line catch (concentrated in coastal areas) is considered. Any comparison between proportions requires care given unknowns of wild population size and catch effort, or even the relative catchability of farmed-origin and wild fish once in the rivers.

The Scottish dataset contains little in the way of stratification by season, however escapes are rare in the catch from the earlier part of the year. (In contrast, the escapes data, aggregated here into years, are described by day of escape.) This is in agreement with findings in Northern Ireland [18] and Norway [28], where escaped farmed salmon tend to enter rivers relatively late in the season. A caveat here is that any seasonal differences in fishing effort by the different methods—in turn concentrated in different sections of the water course—would be confounded with seasonality in appearance of escaped farmed salmon. At shorter timescales beyond the resolution of the Scottish data, a study in Norway [22] reported elevated catch of farmed salmon was detected in fisheries for several weeks after documented escape events; however a considerable ‘background’ rate of farmed salmon of varied size ranges persisted in the catch, suggesting that in the studied regions of Norway, a ‘trickle’ of unreported, small escape events may have been an important source of farmed-origin fish in fishery catches [22].

Given such unknowns in salmon biology and behaviour, we have been flexible in our modelling approach. For example, with comparatively little data indicating how salmon may disperse in the open sea (as opposed to enclosed fjords [24]), it is unclear what the appropriate size of geographical area for study should be. One study [11], finding greater depletion of wild stocks in areas with salmon farms, used data at the river level, a finer geographical scale that was available for our study; however, in another [13], with data aggregated at the regional level, no relationship between prevalence of escapes and reported escapes was found. Our analysis asks a subtly different question: we specifically test for an effect of documented escape events, over and above any baseline differences between districts due to other causes. Possible reasons for an increase in catch after escape events could be misidentification of farmed fish as wild, or increased catch effort following known escape events. Thus, any baseline association between escapes and farmed-origin catch are absorbed into the terms for district- and year-level variation.

Our model results, though in places with terms for lagged escapes significantly related to catch size and proportion of escapes, explained a low proportion of the model variation and showed low robustness to changes in model structure, particularly in the case of the more robust ANOVA models where few terms were found significant. In particular, for proportion of catch reported as escapes, under 10% of deviance was explained by escape lag terms even when non-farmed districts were excluded. Effect sizes were relatively small and with contradictory signs when examined at the district and regional level. This partly stems from relatively complicated models with considerable district-to-district variation, and multiple lag terms for escapes, which were considered necessary due to the long generation time of the species involved. An assumption of both logistic regression and ANOVA is independence of observations. As with many observational studies, there are likely to be uncontrolled grouping variables in our study, such as survey response, individual angler, and sub-district geographical structure.

Despite these caveats of overinterpretation of the model results, some patterns can be ascertained. In district-level models, the proportion of catch reported as of farmed origin was positively associated with local farm escapes in the recent past, but negatively associated at longer time lags. This may be the case if farmed salmon from previous years are more likely to be misidentified as wild fish later. The best-fit model for district levels included escapes of large fish, whereas for regional-level models, all escapes, and with negative coefficients. This is consistent with reported catch (mostly of large fish) being affected more by recent, local fish escapes, with escapes from further back in time being caught over a wider area, and possibly misidentified as wild. The possibility of some form of confounding is also indicated by the difficult-to-explain trout results, where trout catch was found to be positively associated with local escapes of farmed salmon. In addition when only the proportion of farmed fish in the rod-and-line catch was considered, model results were again inconsistent in regional-level models, with negative coefficients.

The historical decline in salmon catch in Scotland fits into the general trend of declining biomass observed in Atlantic salmon across Europe [30]. However, our study relies on secondary data of unknown accuracy, ultimately derived from a large number of questionnaire returns from fisheries (1846 in 2008 alone [14]). Return rate is generally high, though with omissions; for 2008, overall return rate of questionnaires was 93%, with almost all districts with return rates exceeding 80%.

Though we have addressed potential data errors using estimates of potential accuracy from the literature, there are several potential reasons why such parameter estimates may not be appropriate, or even constant between areas. Rod-catch data may poorly estimate occurrence of rod-caught farmed salmon due to both anglers' perceptions and ability to distinguish between salmon types. It can be presumed that given fish of similar possible farm-origin appearance, anglers will be less likely to report these as being of farmed origin when in an area with no history of salmon farming or escapes. Accuracy will also decline over time since escape: particularly for salmon that escape as parr, numbers in the catch statistics may be underestimated [31]. A caveat of this for modelling is that any return of escaping parr caught as adults may be reflected in the model not as escapes, but as a higher total catch. A further concern is that fishing regulations tend to encourage or require catch and release for wild salmon, but retaining escaped farmed salmon is required in some areas, for example the Spey system [32]. This may provide a tasty incentive for characterisation of salmon of unclear origin for a hungry angler. This form of bias would not be so easily identified experimentally by simply testing fishermen for their ability to identify farmed- or wild-origin salmon. Where catch and release occurs for wild salmon, but not for farmed salmon, there is also the potential for the same wild fish to be caught repeatedly, potentially reducing the measured prevalence of farmed salmon, though no data are available on this. Nevertheless, for trout, these sources of bias and misidentification are not present and the analyses should be more robust for this species.

Further possible confounding effects within the data set exist. Catch data primarily pertain to large, adult fish of harvestable size. Therefore, if there are differences in fitness and survivability of farmed and wild-origin fish, these data provide a biased estimate of the prevalence of escapes in smaller, younger fish. Furthermore, except for net and coble, and fixed-engine methods, no record of sampling effort (in terms of time spent fishing) is recorded. Without this, the size of the wild population into which escapes are mingling is difficult to estimate. This is a particular issue when examining data recorded over a longer time series, as changes in catch will reflect not only the biology, but also changes in human habits and industry (for example change in the popularity of angling).

### Conclusions

In summary, in this paper we ask a specific question of the large data sets encompassing salmon and trout catch and of recorded salmon escapes from Scottish salmon farms in the last decade—that is whether a statistically significant effect of the recorded salmon escapes can be found in the catch data, over and above the expected level for the year and district. Our more robust models provide no evidence of depressed catch (either salmon or trout), or firm evidence of elevated prevalence of escapes in the salmon catch in the years immediately following reported escape events.
